# Association between red cell distribution width and HBV viral load and liver function parameters in patients with chronic hepatitis B: a real-world data study

**DOI:** 10.3389/fmed.2026.1780115

**Published:** 2026-05-13

**Authors:** Lei Zhang, Chunhua Fang, Liping Wang, Bin Ran

**Affiliations:** Department of Infectious Diseases, Hefei Third People’s Hospital (Hefei Third Clinical College of Anhui Medical University), Hefei, China

**Keywords:** chronic hepatitis B, liver function, red cell distribution width, retrospective study, viral load

## Abstract

**Background:**

Chronic hepatitis B virus (HBV) infection remains a major global public health challenge. Identifying accessible biomarkers that reflect disease activity is crucial for clinical management. Red cell distribution width (RDW), a parameter routinely reported in complete blood counts, has garnered increasing attention for its role in chronic inflammatory conditions.

**Objective:**

To investigate the correlation between RDW and HBV viral load and liver function parameters in patients with chronic hepatitis B (CHB).

**Methods:**

A total of 120 CHB patients admitted to Hefei Third People’s Hospital (Hefei Third Clinical College of Anhui Medical University) between February 2023 and May 2025 were enrolled as the CHB group, and 70 healthy individuals undergoing routine physical examination during the same period served as controls. Levels of RDW-CV, RDW-SD, HBV DNA viral load, and liver function parameters were measured and compared between groups. Correlations between RDW and these parameters were analyzed.

**Results:**

RDW-CV and RDW-SD levels were significantly higher in the CHB group compared to controls (*t* = 6.784, *P* < 0.001; *t* = 6.105, *P* < 0.001). The high viral load subgroup exhibited significantly elevated RDW-CV and RDW-SD levels relative to the low viral load subgroup (*t* = 4.783, *P* < 0.001; *t* = 3.385, *P* = 0.001). Patients in the immune-active phase had higher RDW-CV and RDW-SD levels than those in the immune-tolerant/inactive phase (*t* = 4.725, *P* < 0.001; *t* = 4.385, *P* < 0.001). Spearman correlation analysis revealed positive correlations of RDW-CV and RDW-SD with HBV DNA load, ALT, AST, TBIL, and APRI, and negative correlations with ALB and PLT. Multivariate logistic regression identified high viral load and elevated ALT as independent factors associated with increased RDW.

**Conclusion:**

RDW is closely associated with viral load and markers of liver injury in CHB patients. These hypothesis-generating findings suggest that RDW warrants further investigation as a potential auxiliary marker for assessing disease activity.

## 1 Introduction

Chronic hepatitis B (CHB), caused by persistent HBV infection, is a chronic inflammatory liver disease affecting approximately 254 million individuals globally, with an estimated 1.1 million annual deaths due to end-stage liver diseases such as cirrhosis, liver failure, and hepatocellular carcinoma, according to the World Health Organization’s 2024 Global Hepatitis Report (1). Disease progression is closely linked to viral replication levels, host immune response, and intrahepatic inflammatory activity. Dynamic monitoring of viral load and accurate assessment of liver injury are critical for treatment decisions and prognosis (2). Red cell distribution width (RDW), a quantitative measure of erythrocyte volume heterogeneity reported by automated hematology analyzers, has traditionally been used for morphological classification and differential diagnosis of anemia (3). Recent studies indicate that RDW is elevated in various inflammation-related diseases, including coronary artery disease, heart failure, chronic kidney disease, and sepsis, and is independently associated with disease severity and clinical outcomes (4). Proposed mechanisms involve inflammatory-mediated suppression of erythropoiesis, erythropoietin resistance, enhanced oxidative stress, and disordered iron metabolism (5). In liver diseases, studies have demonstrated correlations between RDW and Child-Pugh classification, Model for End-Stage Liver Disease (MELD) score, and clinical prognosis in cirrhotic patients, with higher RDW predicting poorer survival (6). In CHB, viral replication triggers immune-mediated hepatocyte damage and release of pro-inflammatory cytokines such as tumor necrosis factor-α (TNF-α) and interleukin-6 (IL-6), which may increase RDW by affecting erythrocyte membrane stability, shortening red cell lifespan, and impairing bone marrow function (7). Additionally, the liver plays a key role in erythropoietin (EPO) metabolism; impaired liver function may alter EPO processing and exacerbate erythropoietic dysfunction (8). Reduced hepatic iron storage and chronic inflammation-induced iron utilization disorders may also contribute to abnormal RDW (9).

Despite growing research on RDW in liver diseases, its clinical application in CHB remains limited. Existing studies have largely focused on end-stage liver diseases such as decompensated cirrhosis or hepatocellular carcinoma, with insufficient investigation into RDW dynamics across different phases of CHB natural history, including immune-tolerant, immune-active, immune-control, and reactivation phases (10). Most reports are limited by small sample sizes and single-center designs, constraints that also apply to the present study despite its relatively larger sample. These studies often lack adequate adjustment for confounding factors such as antiviral treatment history, treatment response, HBV genotype (B/C), HBeAg status, and coexisting conditions like fatty liver or alcoholic liver disease (11). Furthermore, the dose-response relationship and dynamic changes between RDW and virological markers, particularly HBV DNA load, remain unexplored. Our current cross-sectional analysis, while providing correlational evidence, does not fully address this gap; longitudinal studies are needed to evaluate the sensitivity and specificity of RDW in reflecting viral replication and intrahepatic inflammation over time (12). There is a lack of comprehensive analyses evaluating the multivariate associations between RDW and biochemical liver function parameters [e.g., alanine aminotransferase (ALT), aspartate aminotransferase (AST)], bilirubin metabolism, coagulation profiles, and non-invasive fibrosis indices such as the aspartate aminotransferase-to-platelet ratio index (APRI) and the fibrosis index based on four factors (FIB-4) (13). Current guidelines from the International Liver Society, the American Association for the Study of Liver Diseases (AASLD), and the Chinese Guidelines for Prevention and Treatment of Chronic Hepatitis B do not include RDW as a routine monitoring parameter, underscoring the need for higher-quality evidence to support its clinical applicability (14). Furthermore, comparative studies between RDW and established inflammatory markers such as C-reactive protein (CRP) and erythrocyte sedimentation rate (ESR) in CHB patients are scarce, and the incremental value of RDW remains undefined (15). The incremental value of this study relative to existing literature is threefold. First, unlike most previous reports that examined only a single RDW metric, we simultaneously and directly compare the performance of both RDW-CV and RDW-SD, addressing an important gap regarding which parameter may be more informative for CHB. Second, we incorporate multiple non-invasive fibrosis markers (APRI, FIB-4, and RPR) within a unified analytical framework alongside traditional liver function tests, providing a more comprehensive assessment of the RDW–disease activity relationship. Third, we provide multivariable-adjusted estimates that quantify the independent correlates of RDW elevation while explicitly acknowledging residual confounding (e.g., antiviral therapy, HBV genotype) as a limitationiontherapy, HBV genotype) independent correlates of RDW elevation while explicitly aour study highlights specific methodological limitations that future prospective studies should address.

This real-world study aims to systematically examine the correlations between RDW (including RDW-CV and RDW-SD) and HBV DNA load, liver function biochemical parameters, and non-invasive fibrosis scores in CHB patients. Using multivariate regression models and receiver operating characteristic (ROC) curve analysis, we assess independent factors influencing RDW elevation and its predictive performance for disease activity. The findings may provide evidence supporting the potential role of RDW as an auxiliary biomarker for monitoring CHB patients, with practical implications for optimizing clinical management.

## 2 Materials and methods

### 2.1 Study population

This single-center, retrospective cohort study consecutively enrolled 120 patients diagnosed with CHB at the outpatient or inpatient department of our hospital between February 2023 and May 2025, constituting the CHB group. This group included 72 males and 48 females (ratio 1.5:1), with a mean age of 43.15 years (SD 12.87; range 18–70 years), consistent with the epidemiological profile of CHB in China. For comparison, 70 individuals undergoing routine health examinations at our center during the same period were enrolled as healthy controls. Controls were frequency-matched by gender and age (no significant intergroup difference, *p* > 0.05). All control subjects tested negative for hepatitis B surface antigen, hepatitis C antibody, and human immunodeficiency virus antibody, and had normal liver function test results.

Sample size calculation: Based on preliminary data and published literature, we estimated a standardized effect size (Cohen’s *d*) of approximately 0.8 for RDW differences between groups. Using PASS 2021 software with a two-sided α of 0.05 and 80% power, the required sample size per group was calculated using the formula for independent samples: *n* = 2 × (Z_1_-α/_2_ + Z_1_-β)^2^ × σ^2^/δ^2^, where Z_1_-α/_2_ = 1.96, Z_1_-β = 0.84, σ is the estimated standard deviation, and δ is the expected difference. This yielded a minimum of 56 subjects per group. Accounting for potential data missingness or exclusion in retrospective studies, the final sample sizes (CHB group: *n* = 120; control group: *n* = 70) exceeded this requirement, ensuring sufficient statistical reliability.

### 2.2 Sample collection and preparation

All data were retrospectively extracted from the integrated electronic medical records and laboratory information systems of our institution. No new samples were collected specifically for this study; all laboratory measurements were performed as part of routine clinical care during the study period. For complete blood count analysis, fasting venous blood (2 mL) was collected in EDTA-K2 tubes and analyzed within 2 h. Samples were processed using a Sysmex XN-9000 automated hematology analyzer (Sysmex Corporation, Japan) with calibrators, controls, and reagents. For serum biochemical analysis, fasting venous blood (5 mL) was collected in serum separator tubes, allowed to clot for 30 min at room temperature, and centrifuged at 3,000 rpm (1,500 × g) for 10 min at 4°C. Serum was separated and analyzed within 4 h using a Cobas 8,000 automated biochemical analyzer (Roche Diagnostics, Switzerland). For HBV DNA quantification, serum samples were processed using the Cobas AmpliPrep automated nucleic extraction system and Cobas TaqMan 48 real-time PCR system (Roche Diagnostics). All laboratory procedures followed manufacturer instructions and institutional standard operating procedures.

### 2.3 Inclusion and exclusion criteria

Inclusion criteria for the CHB group:

(1) Age ≥ 18 years;
(2) Diagnosis meeting the 2019 Chinese Guidelines for Prevention and Treatment of Chronic Hepatitis B (HBsAg positivity for > 6 months);
(3) Complete clinical records and key laboratory data for this study.

Inclusion criteria for the control group:

(1) Age ≥ 18 years;
(2) Negative for HBsAg, HCV antibody, and HIV antibody;
(3) Normal liver function tests (ALT, AST, total bilirubin, albumin) per institutional reference ranges;
(4) No history of chronic liver disease, hematological disorders, or autoimmune diseases.

Exclusion criteria (both groups):

(1) Coinfection with other hepatitis viruses (A, C, D, E) or HIV;
(2) Clinical, imaging (e.g., abdominal ultrasound, CT), or histopathological diagnosis of decompensated cirrhosis, hepatocellular carcinoma, or any other malignancy;
(3) History of blood transfusion or major surgery within 3 months prior to enrollment;
(4) Hematological diseases significantly affecting erythrocyte parameters and RDW (e.g., iron deficiency anemia, thalassemia, megaloblastic anemia, myelodysplastic syndrome) or autoimmune diseases associated with secondary anemia (e.g., systemic lupus erythematosus, rheumatoid arthritis);
(5) Pregnancy or lactation;
(6) Major organ dysfunction, including NYHA class III–IV heart failure, severe renal impairment (eGFR < 30 mL/min/1.73 m^2^), or severe post-stroke disability.

### 2.4 Equipment and assays

All laboratory tests were performed in our central laboratory under standardized operating procedures and strict quality control.

(1) Complete blood count and RDW: Measured using a Sysmex XN-9000 automated hematology analyzer (Sysmex Corporation, Japan) with calibrators, controls, and reagents. Fasting venous blood (2 mL) was collected in EDTA-K2 tubes and analyzed within 2 h. RDW-CV and RDW-SD were determined via DC impedance and flow cytometry with fluorescent staining. Internal quality control was performed daily, and external quality assurance was maintained through programs organized by the National Center for Clinical Laboratories.
(2) Liver function tests: Measured using a Cobas 8000 automated biochemical analyzer (Roche Diagnostics, Switzerland) with reagents. Serum was separated from 5 mL fasting venous blood after centrifugation. ALT and AST were measured by the IFCC-recommended kinetic method; total bilirubin by the diazo method; albumin by the bromocresol green method. All procedures followed manufacturer instructions and laboratory protocols.
(3) HBV DNA quantification: Performed using the Cobas AmpliPrep automated nucleic extraction system and Cobas TaqMan 48 real-time PCR system (Roche Diagnostics) with the Cobas TaqMan HBV Test v2.0 kit. The detection range was 20–1.7 × 10^8^ IU/mL. Serum samples were preprocessed, extracted, amplified, and quantified per protocol. Viral load was reported in IU/mL and log10-transformed for statistical analysis.

### 2.5 Study procedures

(1) Retrospective data collection: Two trained researchers independently extracted baseline data from integrated electronic medical records and laboratory information systems for all eligible subjects. Data included demographics (age, gender), clinical diagnosis, and laboratory results (complete blood count, liver function tests, HBV DNA) obtained within the same visit or within a 1-week interval. Data were cross-checked, with discrepancies resolved by a senior researcher. Cases missing key variables (e.g., RDW, HBV DNA, ALT) were excluded.
(2) Subgroup definitions and analysis: To explore RDW associations with HBV disease status, CHB patients were subdivided:

Based on baseline HBV DNA level, using 2 × 10^4^ IU/mL as cutoff: high vs. low viral load subgroups.

Based on the 2018 American Association for the Study of Liver Diseases (AASLD) guidelines for chronic hepatitis B, CHB patients were categorized according to disease activity. The immune-active phase was defined as patients with ALT levels exceeding the institutional upper limit of normal (ULN; male: 35 U/L, female: 25 U/L) and HBV DNA levels > 2,000 IU/mL (for HBeAg-negative) or > 20,000 IU/mL (for HBeAg-positive). The immune-tolerant/inactive phase was defined as patients with ALT ≤ ULN and low HBV DNA levels (< 2,000 IU/mL for HBeAg-negative; < 20,000 IU/mL for HBeAg-positive), consistent with AASLD criteria.

Inter-subgroup differences in RDW and other parameters were compared.

(3) Correlation analysis: Univariate analyses compared RDW levels between groups and subgroups. Spearman correlation assessed relationships between RDW and continuous variables: log10 HBV DNA, ALT, AST, TBIL, ALB, APRI, FIB-4, and RPR. Non-parametric methods were used for non-normally distributed data.

### 2.6 Outcome measures

The following eight core parameters were analyzed:

(1) RDW-CV (%): Primary RDW parameter, calculated as (SD of erythrocyte volume/mean corpuscular volume) × 100%.
(2) RDW-SD (fL): Absolute measure of erythrocyte volume dispersion.
(3) HBV DNA (log10 IU/mL): Direct quantitative marker of viral replication.
(4) ALT (U/L): Sensitive marker of hepatocellular inflammation.
(5) AST (U/L): Marker of hepatocellular injury, often analyzed with ALT.
(6) Total bilirubin (TBIL, μmol/L): Reflects hepatic bilirubin metabolism.
(7) Albumin (ALB, g/L): Indicator of hepatic synthetic function.
(8) APRI: Calculated as [(AST/AST ULN)/platelet count (10^9^/L)]; AST ULN = 40 U/L. Higher values indicate greater fibrosis likelihood. (9) FIB-4: Calculated as [age (years) × AST (U/L)]/[platelet count (10^9^/L) × √ALT (U/L)]. (10) RPR (RDW-to-platelet ratio): Calculated as RDW-CV (%)/platelet count (10^9^/L).

### 2.7 Statistical analysis

Analyses were performed using IBM SPSS Statistics 26.0. Continuous variables were tested for normality via Kolmogorov-Smirnov test and histograms. Normally distributed data are presented as mean ± SD and compared using independent *t*-tests; non-normal data as median (IQR) compared with Mann-Whitney U test. Categorical variables are described as frequencies (%) and compared with chi-square or Fisher’s exact test.

Spearman rank correlation evaluated associations between RDW and continuous laboratory parameters. Multivariate binary logistic regression identified independent clinical factors associated with elevated RDW, including viral load (high/low), ALT status (immune-active/not), albumin (< 40 g/L/not), platelet count (< 150 × 10^9^/L/not), with adjustment for age and gender. Odds ratios (OR) and 95% confidence intervals (CI) were calculated.

ROC curves assessed the predictive value of RDW for high viral load or significant liver injury, determining AUC, optimal cutoff, sensitivity, and specificity. All tests were two-sided; *p* < 0.05 indicated statistical significance.

## 3 Results

### 3.1 Comparison of baseline characteristics

No significant differences were observed in demographic characteristics, including age (*t* = 0.684, *P* = 0.495) and gender distribution (χ^2^ = 0.612, *P* = 0.434), between the chronic hepatitis B (CHB) group and the healthy control group. Compared to the control group, CHB patients exhibited significantly elevated liver function parameters: alanine aminotransferase (ALT) (*t* = 7.583, *P* < 0.001), aspartate aminotransferase (AST) (*t* = 6.927, *P* < 0.001), and total bilirubin (TBIL) (*t* = 6.128, *P* < 0.001), alongside significantly reduced albumin (ALB) levels (*t* = -8.392, *P* < 0.001). Hematological analysis revealed higher red cell distribution width coefficient of variation (RDW-CV) (*t* = 6.784, *P* < 0.001) and red cell distribution width standard deviation (RDW-SD) (*t* = 6.105, *P* < 0.001), but lower platelet count (PLT) (*t* = -8.531, *P* < 0.001) in the CHB group. Additionally, the aspartate aminotransferase-to-platelet ratio index (APRI) was significantly higher in the CHB group (*t* = 8.264, *P* < 0.001) (see Table 1).

**TABLE 1:** Comparison of baseline characteristics between the chronic hepatitis B group and the healthy control group.

Parameter	CHB Group (*n* = 120)	Healthy Control Group (*n* = 70)	Statistic	*P*-value
Demographics				
Age (years)	43.15 ± 12.87	41.89 ± 11.24	*t* = 0.684	0.495
Male, n (%)	72 (60.00)	38 (54.29)	χ^2^ = 0.612	0.434
Virological parameter				
HBV DNA (log10 IU/mL)	4.73 ± 1.85	–	–	–
Liver function parameters				
ALT (U/L)	58.36 ± 45.17	21.43 ± 6.85	*t* = 7.583	< 0.001
AST (U/L)	49.82 ± 36.94	22.15 ± 5.12	*t* = 6.927	< 0.001
TBIL (μmol/L)	18.95 ± 9.63	12.41 ± 3.57	*t* = 6.128	< 0.001
ALB (g/L)	41.26 ± 5.37	46.83 ± 3.15	*t* = -8.392	< 0.001
Hematological parameters				
RDW-CV (%)	14.28 ± 1.65	12.95 ± 0.78	*t* = 6.784	< 0.001
RDW-SD (fL)	45.36 ± 5.92	41.05 ± 3.14	*t* = 6.105	< 0.001
PLT (× 10^9^/L)	156.32 ± 51.47	218.64 ± 47.26	*t* = -8.531	< 0.001
Calculated index				
APRI	0.83 ± 0.71	0.21 ± 0.05	*t* = 8.264	< 0.001

### 3.2 Comparison between different viral load subgroups

Subgroup analysis based on viral load demonstrated significantly higher HBV DNA levels in the high viral load subgroup compared to the low viral load subgroup (*t* = 18.527, *P* < 0.001). Regarding liver function, the high viral load subgroup exhibited significantly elevated ALT (*t* = 5.927, *P* < 0.001), AST (*t* = 5.384, *P* < 0.001), and TBIL (*t* = 3.472, *P* = 0.001) levels. No significant difference in ALB was observed between subgroups (*t* = –1.583, *P* = 0.116). Hematologically, both RDW-CV (*t* = 4.783, *P* < 0.001) and RDW-SD (*t* = 3.385, *P* = 0.001) were higher in the high viral load subgroup, whereas PLT count did not differ significantly (*t* = –1.537, *P* = 0.127). APRI, FIB-4, and RPR were also significantly higher in the high viral load subgroup (APRI: *t* = 5.826, *P* < 0.001; FIB-4: *t* = 5.312, *P* < 0.001; RPR: *t* = 6.158, *P* < 0.001) (see Table 2).

**TABLE 2:** Comparison of parameters between different viral load subgroups in chronic hepatitis B.

Parameter	High viral load group (*n* = 65)	Low viral load group (*n* = 55)	Statistic	*P*-value
Virological parameter				
HBV DNA (log10 IU/mL)	6.12 ± 0.89	3.11 ± 0.92	*t* = 18.527	< 0.001
Liver function parameters				
ALT (U/L)	75.83 ± 48.25	37.82 ± 25.41	*t* = 5.927	< 0.001
AST (U/L)	63.15 ± 39.47	34.16 ± 21.58	*t* = 5.384	< 0.001
TBIL (μmol/L)	21.47 ± 10.35	15.98 ± 7.24	*t* = 3.472	0.001
ALB (g/L)	40.58 ± 5.41	42.05 ± 5.18	*t* = –1.583	0.116
Hematological parameters				
RDW-CV (%)	14.87 ± 1.72	13.58 ± 1.25	*t* = 4.783	< 0.001
RDW-SD (fL)	46.92 ± 6.15	43.52 ± 4.87	*t* = 3.385	0.001
PLT (× 10^9^/L)	149.76 ± 53.62	163.85 ± 48.35	*t* = –1.537	0.127
Calculated index				
APRI	1.12 ± 0.85	0.49 ± 0.32	*t* = 5.826	< 0.001

### 3.3 Comparison between different disease activity subgroups

Compared to patients in the immune-tolerant or inactive phase, those in the immune-active phase displayed significantly higher ALT (*t* = 13.257, *P* < 0.001), AST (*t* = 11.384, *P* < 0.001), and TBIL (*t* = 4.835, *P* < 0.001) levels, alongside significantly lower ALB levels (*t* = –2.625, *P* = 0.010). No significant difference in HBV DNA was observed between subgroups (*t* = 1.135, *P* = 0.259). The immune-active phase was associated with higher RDW-CV (*t* = 4.725, *P* < 0.001) and RDW-SD (*t* = 4.385, *P* < 0.001), but lower PLT count (*t* = –2.683, *P* = 0.008). APRI, FIB-4, and RPR were also significantly elevated in the immune-active phase subgroup (APRI: *t* = 8.157, *P* < 0.001; FIB-4: *t* = 7.643, *P* < 0.001; RPR: *t* = 8.924, *P* < 0.001) (see Table 3).

**TABLE 3:** Comparison of parameters between different disease activity subgroups in chronic hepatitis B.

Parameter	Immune-active phase (*n* = 52)	Immune-tolerant/Inactive phase (*n* = 68)	Statistic	*P*-value
Virological parameter				
HBV DNA (log10 IU/mL)	4.95 ± 1.92	4.57 ± 1.78	*t* = 1.135	0.259
Liver function parameters				
ALT (U/L)	95.26 ± 38.45	32.15 ± 12.38	*t* = 13.257	< 0.001
AST (U/L)	78.43 ± 35.26	29.45 ± 14.72	*t* = 11.384	< 0.001
TBIL (μmol/L)	23.18 ± 10.14	15.82 ± 7.35	*t* = 4.835	< 0.001
ALB (g/L)	39.85 ± 5.62	42.28 ± 4.85	*t* = –2.625	0.01
Hematological parameters				
RDW-CV (%)	15.02 ± 1.78	13.72 ± 1.28	*t* = 4.725	< 0.001
RDW-SD (fL)	47.85 ± 6.32	43.42 ± 4.65	*t* = 4.385	< 0.001
PLT (× 10^9^/L)	142.58 ± 49.73	166.82 ± 49.15	*t* = –2.683	0.008
Calculated index				
APRI	1.35 ± 0.92	0.45 ± 0.25	*t* = 8.157	< 0.001

### 3.4 Correlation analysis of red cell distribution width with various parameters

**FIGURE 1:**
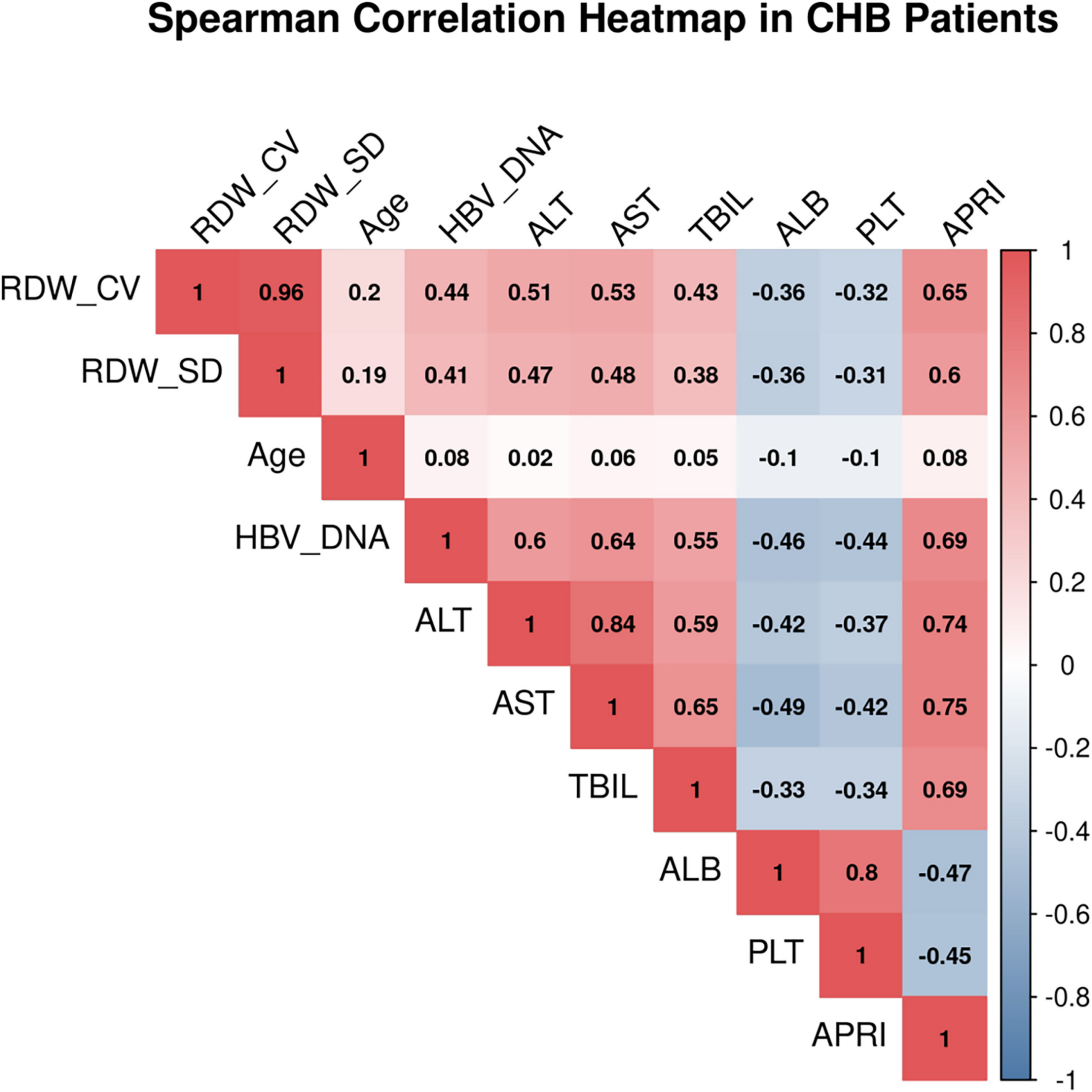
Spearman correlation heatmap in CHB patients.

**FIGURE 2:**
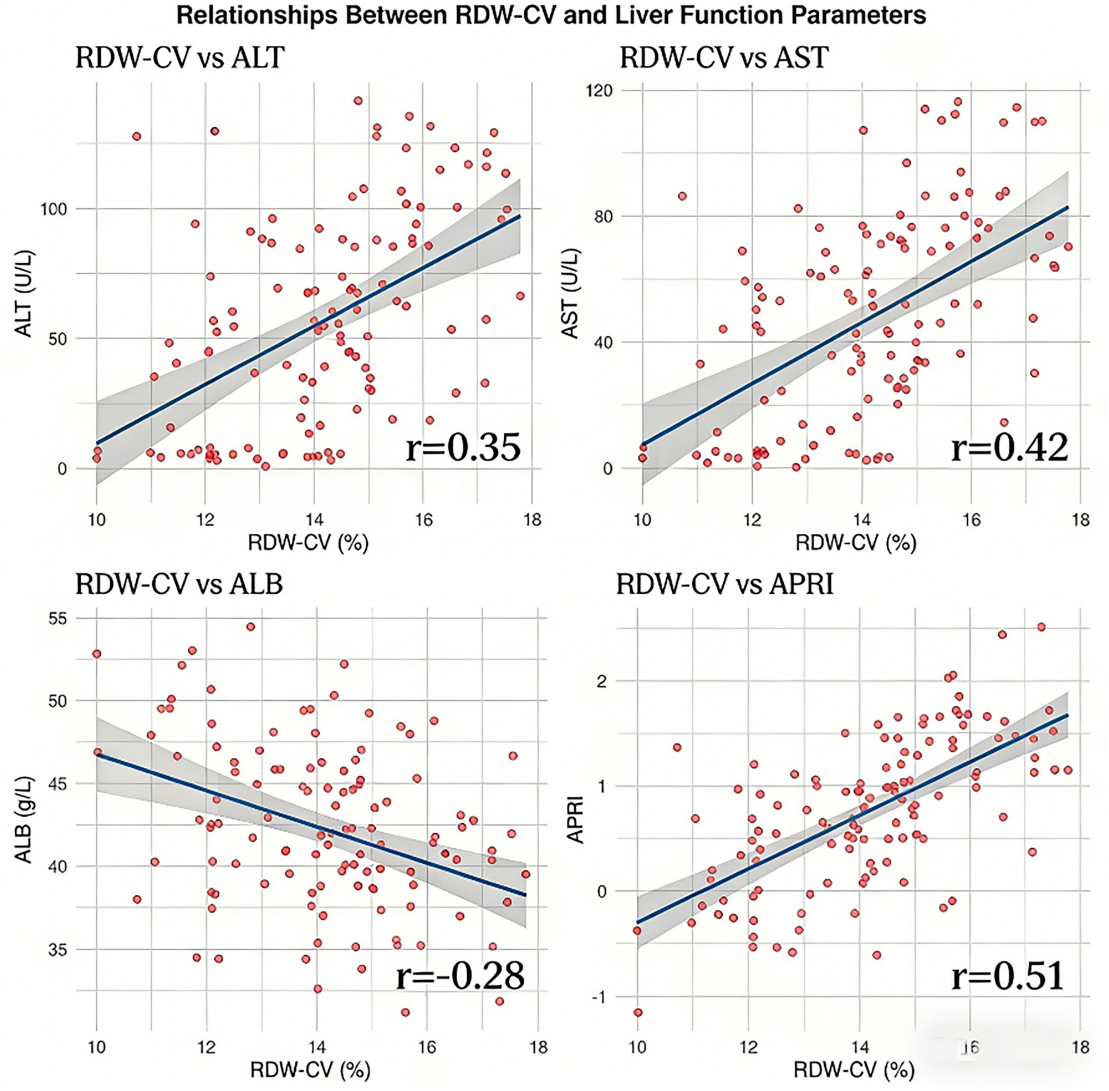
Relationships between RDw-CV and liver function parameters.

Spearman correlation analysis revealed no significant correlation between either RDW-CV or RDW-SD and patient age (*P* > 0.05). Both RDW-CV and RDW-SD showed significant positive correlations with HBV DNA (*P* < 0.001), ALT (*P* < 0.001), AST (*P* < 0.001), TBIL (*P* < 0.001), APRI (*P* < 0.001), FIB-4 (*P* < 0.001), and RPR (*P* < 0.001). Significant negative correlations were observed with ALB (*P* < 0.001) and PLT (*P* < 0.05) (see Figures 1, 2).

### 3.5 Multivariate logistic regression analysis of factors influencing high red cell distribution width

Multivariate logistic regression was performed with the presence of high red cell distribution width status as the dependent variable. Independent variables were defined as follows: high viral load (yes = 1, no = 0), high ALT (immune-active phase) (yes = 1, no = 0), low ALB (< 40 g/L) (yes = 1, no = 0), and low PLT (< 150 × 10^9^/L) (yes = 1, no = 0). The analysis identified high viral load (OR = 3.08, 95% CI: 1.46–6.51, *P* = 0.003) and high ALT (immune-active phase) (OR = 4.21, 95% CI: 1.94–9.15, *P* < 0.001) as independent risk factors for high red cell distribution width in CHB patients. Low ALB (OR = 2.19, 95% CI: 1.00–4.80, *P* = 0.051) and low PLT (OR = 1.92, 95% CI: 0.85–4.33, *P* = 0.116) were not independently associated with high RDW status (see Table 4).

**TABLE 4:** Logistic regression analysis of factors influencing high red cell distribution width in chronic hepatitis B patients.

Variable	β -value	SE	Wald χ ^2^	OR (95% CI)	*P*-value
High viral load	1.125	0.382	8.672	3.08 (1.46, 6.51)	0.003
High ALT (Immune-Active)	1.437	0.396	13.158	4.21 (1.94, 9.15)	< 0.001
Low ALB (< 40 g/L)	0.783	0.401	3.814	2.19 (1.00, 4.80)	0.051
Low PLT (< 150 × 10^9^/L)	0.652	0.415	2.468	1.92 (0.85, 4.33)	0.116

### 3.6 Predictive value of red cell distribution width for disease status

**FIGURE 3:**
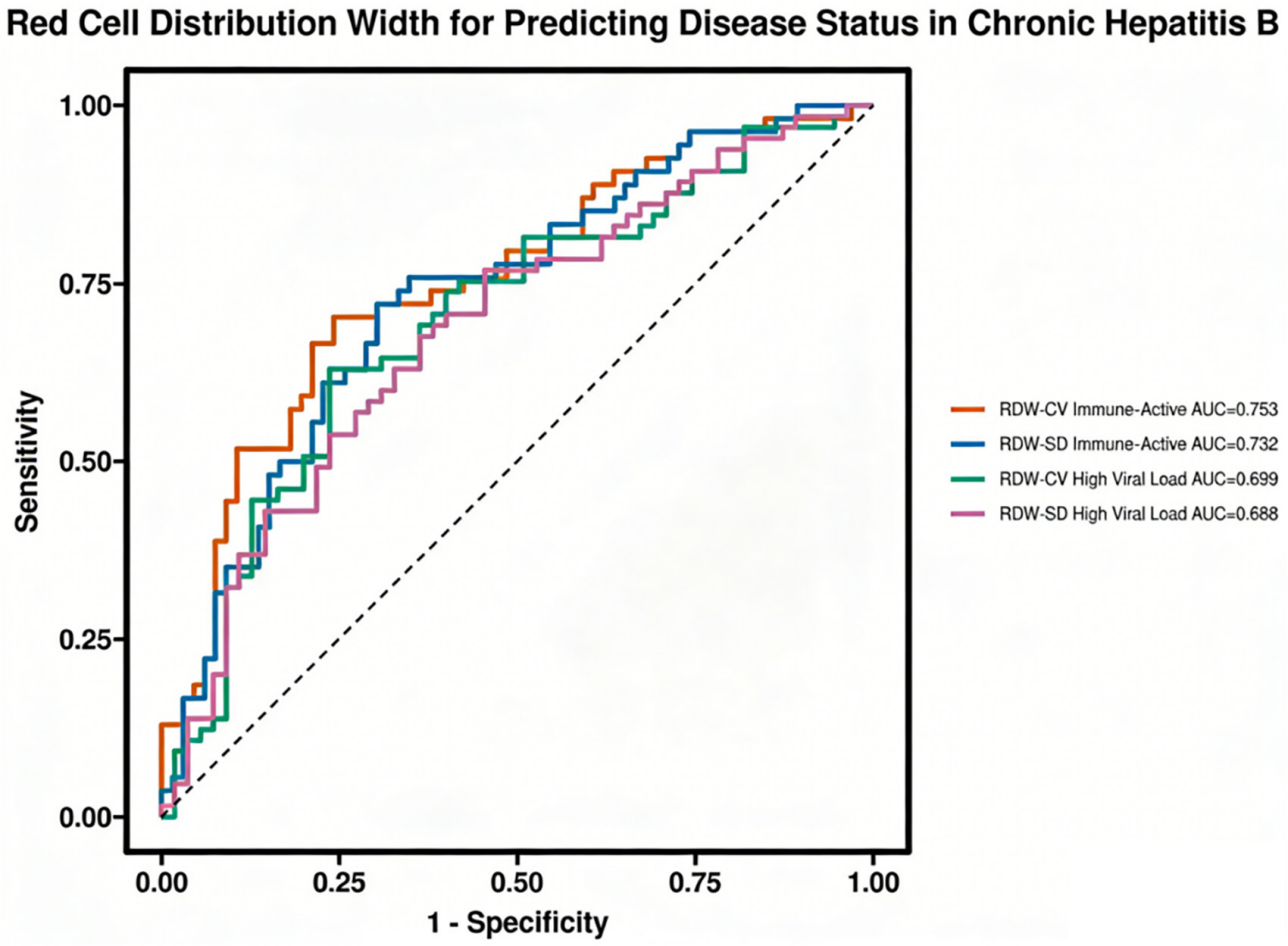
ROC Curve analysis of red cell distribution width for predicting disease status in chronic hepatitis B.

ROC curve analysis was performed to evaluate the predictive utility of red cell distribution width parameters for disease status in chronic hepatitis B. Both RDW-CV and RDW-SD demonstrated moderate predictive performance for identifying the immune-active phase, with AUC values of 0.754 and 0.732, respectively (*P* < 0.05 for both). DeLong test revealed no significant difference between the AUC of RDW-CV (0.754) and RDW-SD (0.732) for predicting immune-active phase (*P* = 0.432). For comparison, the AUC for ALT alone in predicting immune-active phase was 0.892 (95% CI: 0.835–0.949), and for APRI was 0.845 (95% CI: 0.779–0.911), indicating that established markers outperformed RDW in this cohort. Similarly, for predicting high viral load, the AUCs for RDW-CV (0.700) and RDW-SD (0.688) did not differ significantly (*P* = 0.586). These parameters also showed significant predictive capability for high viral load status (*P* < 0.05), though with slightly lower AUC estimates. Optimal cutoff values were established to maximize sensitivity and specificity across all comparisons. Notably, RDW-SD exhibited higher sensitivity for predicting high viral load, while RDW-CV demonstrated greater specificity in both clinical categories. All AUC values were statistically significant (*P* < 0.05), collectively supporting the potential diagnostic utility of RDW parameters in chronic hepatitis B management (see Table 5 and Figure 3).

**TABLE 5:** ROC curve analysis and DeLong test comparison of red cell distribution width parameters for predicting disease status in chronic hepatitis B.

Indicator	AUC	Cutoff	Sensitivity	Specificity	AUC_Formatted
RDW-CV Immune-active phase	0.7539282	14.50687	0.7037037	0.7575758	0.754 (0.666–0.842)
RDW-SD Immune-active phase	0.7323232	45.75696	0.7222222	0.6969697	0.732 (0.642–0.823)
RDW-CV high viral load	0.6998601	14.50687	0.6307692	0.7636364	0.700 (0.605–0.795)
RDW-SD high viral load	0.6881119	44.14182	0.7692308	0.5454545	0.688 (0.593–0.784)

## 4 Discussion

This retrospective study aimed to investigate the association between red cell distribution width (RDW)—an easily accessible parameter from routine blood tests—and core disease features, namely viral replication levels and liver injury status, in patients with chronic hepatitis B (CHB) (16). Our data confirmed that RDW levels were significantly elevated in CHB patients compared to healthy individuals. More importantly, among CHB patients, RDW levels not only showed significant positive correlations with serum HBV DNA load and hepatocellular injury markers such as ALT and AST, but were also more pronounced in subgroups with higher viral load or those in the immune-active phase (17). These findings suggest that RDW may not merely be an indicator of anemia or erythrocyte heterogeneity; its alterations may reflect intrinsic links with the virological status and hepatic inflammatory activity in CHB patients.

Baseline comparisons revealed that, in addition to the expected abnormal liver function, CHB patients had significantly higher RDW values than healthy controls (*P* < 0.001 for both RDW-CV and RDW-SD). This observation is consistent with recent findings reported in the literature, including a study by Ramzi et al. that demonstrated elevated RDW in patients with chronic liver diseases (18). Several mechanisms have been proposed for RDW elevation in chronic liver disease, including inflammation-mediated erythropoiesis suppression, oxidative stress, and impaired erythropoietin metabolism (19). While our study was not designed to test these mechanisms, our observed correlations are consistent with this framework. Direct experimental validation is needed.

Stratified analysis based on viral load showed that patients with high viral replication had higher RDW levels. However, patients in the high viral load subgroup also exhibited higher ALT, AST, and APRI levels, suggesting that liver fibrosis severity may mediate the association between viral load and RDW. Our current analysis did not formally test this mediating effect, which represents a logical limitation when inferring that RDW directly reflects viral load. Future mediation analyses are warranted to delineate whether RDW elevation is primarily driven by viral burden or by fibrosis severity. Nonetheless, the correlation suggests that the viral burden itself, or its accompanying immune milieu, may influence erythrocyte homeostasis. High viral load typically reflects active replication and potential immune pressure. Persistent antigenic stimulation may drive a chronic, low-grade systemic inflammatory response, which is considered one of the core pathophysiological mechanisms leading to increased RDW (20). Therefore, RDW may serve as a peripheral blood marker indirectly reflecting the virus-related inflammatory burden in CHB patients.

Further stratification based on ALT levels into disease activity status revealed that patients in the immune-active phase (elevated ALT) had significantly higher RDW levels than those in the immune-tolerant or inactive phase. This reinforces the view that RDW is closely associated with hepatic inflammation (21). Substances released from necrotic hepatocytes and locally infiltrating immune cells can exacerbate inflammatory cascades, and this liver-specific inflammation may systemically affect the hematopoietic microenvironment. Notably, in this study, RDW also showed a strong correlation with APRI, a non-invasive fibrosis index, suggesting that elevated RDW may be linked not only to immediate inflammation but also to the progression of liver fibrosis due to chronic injury (22).

Spearman correlation analysis provided support for these group differences at the continuous variable level, clearly demonstrating positive correlations between RDW and log10-transformed HBV DNA load, transaminases, and bilirubin, as well as a negative correlation with albumin. This pattern of association aligns with the “inflammation–injury” pathophysiological framework of chronic liver disease. Exploratory multivariate logistic regression analysis identified high viral load and elevated ALT as factors independently associated with RDW elevation (23, 24). Given that RDW is a readily accessible parameter, the clinical utility of using these factors to predict RDW status is limited; rather, this analysis provides hypothesis-generating insights that changes in RDW in CHB patients may be influenced by both viral factors (replication level) and host factors (hepatic inflammatory response).

ROC curve analysis showed that RDW had only moderate discriminatory ability for identifying immune-active phase (AUC 0.732–0.754) and high viral load (AUC 0.688–0.700). These values indicate suboptimal standalone diagnostic performance. Given that CHB staging already relies on readily available and better-performing markers [e.g., ALT (AUC 0.892) and APRI (AUC 0.845)], the incremental clinical value of RDW is limited. At most, RDW might serve a complementary, hypothesis-generating role when interpreted alongside conventional parameters—not as a replacement or adjunct with proven utility (25, 26).

This study has several limitations. First, the retrospective design cannot establish a causal relationship between RDW and CHB progression. Second, we were unable to capture several important potential confounders, including detailed antiviral therapy status (type, duration, adherence), HBV genotype (B vs. C), HBeAg status, and metabolic comorbidities such as non-alcoholic fatty liver disease and alcohol consumption. Although patients who initiated or changed antiviral therapy within the preceding 6 months were excluded to minimize confounding, residual confounding from these unmeasured variables may still influence our findings. Third, as a single-center study, generalizability is limited, and validation in multicenter prospective cohorts is needed. Information on potential confounders (antiviral therapy history, HBV genotype, fatty liver disease) was not collected. Fourth, we did not assess long-term clinical outcomes (fibrosis progression, cirrhosis, mortality), which limits translational value. Future prospective studies should evaluate whether RDW predicts clinically meaningful outcomes and should incorporate dynamic monitoring of RDW during antiviral therapy alongside histological assessments to address dose-response relationships that remain unclear from cross-sectional data.

In conclusion, this single-center real-world study demonstrated that red cell distribution width is significantly correlated with viral load and markers of liver injury in patients with chronic hepatitis B, with more pronounced elevation in those with higher disease activity. As an inexpensive, routinely measured blood parameter, RDW may provide additional reference information for comprehensively assessing inflammatory status and disease activity in CHB patients.

## Data Availability

The datasets presented in this article are not readily available because the datasets generated and analyzed during the current study are not publicly available due to patient privacy and confidentiality considerations, but are available from the corresponding author on reasonable request and with appropriate ethical approval. Requests to access the datasets should be directed to Lei Zhang, 18164487798@163.com.
